# Synergistic effect of acidity and extraframework position in faujasite on renewable *p*-xylene production

**DOI:** 10.1098/rsos.172471

**Published:** 2018-05-23

**Authors:** Eyas Mahmoud

**Affiliations:** 1 Department of Chemical and Petroleum Engineering, United Arab Emirates University, Al-Ain, UAE; 2 Department of Chemical and Biomolecular Engineering, University of Delaware, Newark, DE, USA

**Keywords:** biomass conversion, DMF, ethylene, zeolite, Diels–Alder cycloaddition

## Abstract

*p*-Xylene is a commodity chemical used for the manufacture of plastic bottles and textiles. For the biomass-based route from 2,5-dimethylfuran (DMF) and ethylene, the properties of the catalyst such as acidity effect, product selectivity and catalyst activity play an important role. To determine the effect of acidity and extraframework position in faujasite zeolite on *p*-xylene selectivity, type Y (Si/Al = 40 and Si/Al = 2.55) and X (Si/Al = 1.25) zeolites containing the extraframework Lewis acids Na+, K+, Li+, Ag+ and Cu+, and a Brønsted acid-containing zeolite, HY (Si/Al = 40), were prepared and tested for *p*-xylene production under solvent-free conditions and at low conversions (less than 35%). Here it is reported that NaX zeolite catalyses DMF and ethylene conversion to *p*-xylene with 91% selectivity at 30% conversion, which is better than the 25% *p*-xylene selectivity obtained when using HY at similar conversion. ANOVA was used to show that there is a synergistic effect between acidity and extraframework position on the rate of *p*-xylene production. At 7% DMF conversion, Lewis acids were more selective than the Brønsted acid tested (50 versus 30% *p*-xylene selectivity). *p*-Xylene selectivity is optimal when using Lewis acids with moderate acidity and extraframework positions located in the faujasite supercage (sites II and III).

## 1. Introduction

**Figure 1.**
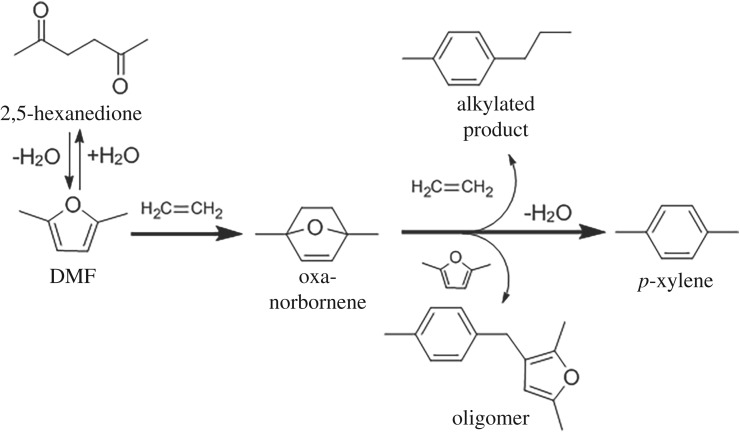
Synthesis of *p*-xylene from DMF and ethylene.

*p*-Xylene is an important commodity chemical used to produce terephthalic acid and polyethylene terephthalate (PET). PET is produced from petroleum at a rate of over 31 million tons per year for plastic bottle and textile manufacture [1]. As an alternative to the conventional petrochemical route, a biomass-based route to *p*-xylene was demonstrated via the Diels–Alder cycloaddition of 2,5-dimethylfuran (DMF) and ethylene to form 1,4-dimethyl-7-oxabicyclo[2.2.1]hept-2-ene (oxanorbornene), which is subsequently dehydrated to *p*-xylene over acid catalysts (figure 1) [2]. The economics and environmental impact of this process are affected by product selectivity and the solvent. For example, sensitivity analysis was used to show that a 15% decrease in *p*-xylene selectivity increases the minimum *p*-xylene cost by 21.7%. The use of *n*-heptane as a solvent for the process adds approximately 4% of the estimated *p*-xylene cost and contributes to marine and freshwater ecotoxicity as well as fossil fuel depletion [3–5]. Three main side reactions decrease *p*-xylene selectivity: hydrolysis of DMF to 2,5-hexanedione, alkylation of reaction intermediates by ethylene and Friedel–Crafts-type reactions of dehydration intermediates which result in oligomer formation (figure 1) [6]. The effect of the properties of the catalyst such as acidity and of zeolite catalysts such as framework type, acidity, mesoporosity on *p*-xylene yield and selectivity in *n*-heptane solvent has been studied [7–10]. Solvent effects have been studied by microkinetic modelling and experiment, and revealed a hydrophobic effect in HY zeolite [11]. Density functional theory (DFT) has provided insight into the reaction mechanism, kinetic regime change and the effect of active site type on catalyst activity [12–13]. Although this biomass-based route to *p*-xylene has been researched intensively, the solvent-free route to *p*-xylene has not received much attention despite the decreased *p*-xylene cost and environmental impact. An experimental study on the effect of active site type and extraframework position in the faujasite zeolite under solvent-free conditions has not been reported and aids computational studies.

The active site in the zeolite catalyst and solvent are critical factors affecting *p*-xylene selectivity and catalyst activity. The active site and framework type of faujasite (X or Y) affect the basicity and the adsorptive properties of the catalyst [14–17]. When comparing Brønsted acid sites in different zeolite frameworks under solvent-free conditions (pure DMF), HY (FAU, Si/Al = 2.6) catalyses the reaction with better selectivity than H-beta and H-ZSM-5 zeolites (52% *p*-xylene selectivity at 11% DMF conversion at 573 K) [2]. In *n*-heptane, H-beta is more selective for *p*-xylene production than HY [18]. Varying DMF concentration from 0.44 to 2.56 mM (0.36 wt.% DMF) at 473 K in *n*-heptane decreases *p*-xylene selectivity from 80% to 55% and increases products of Friedel–Crafts-type reactions from 18 to over 50% for reactions catalysed by HY [19]. Comparing framework Lewis acid active sites, Zr-, Sn- and Ti, in zeolite beta to H-beta for reactions at 523 K, provide a higher selectivity (greater than 70%) in *n*-heptane solvent [20]. DFT and hybrid quantum mechanics/molecular mechanics calculations on alkali-exchanged Y and HY zeolites show that HY is more effective at catalysing the aromatization of the oxanorbornene than alkali-Y (figure 1) [21]. DFT calculations on a high-silica alkali-exchanged faujasite model representing isolated active cation sites and a low-silica alkali-exchanged faujasite in which the reaction involves several cations in the proximity reveals a significant synergetic cooperative effect of the ensemble of cations in the faujasite supercage on the DAC/D reaction, with KY being the most active catalyst tested [22,23]. An experimental study of the effect of acidity and extraframework position in faujasite on solvent-free *p*-xylene production has not been reported.

To examine the effect of active site and extraframework position in faujasite on *p*-xylene selectivity under solvent-free conditions, type Y (Si/Al = 2.55) and X (Si/Al = 1.25) zeolites containing the extraframework Lewis acids Na+, K+, Li+, Ag+ and Cu+, and a Brønsted acid-containing zeolite, HY, were prepared by ion exchange and tested for *p*-xylene production at low conversion (less than 35%). NaX catalyses *p*-xylene production with 96% selectivity at 10% DMF conversion at 523 K. High selectivity to *p*-xylene (greater than 90%) is maintained at conversions greater than 30%. Lewis acidic faujasite was found to be more selective than Brønsted acidic faujasite. Analysis of variance (ANOVA) of the reaction results supports the hypothesis of a synergistic effect as found by DFT calculations [22,23]. The highest selectivity observed is associated with zeolite X where ions take on positions II and III in the faujasite supercage.

## 2. Material and methods

### 2.1. Catalyst preparation and characterization

The sodium form of zeolites Y and X with SiO_2_/Al_2_O_3_ mole ratios of 40, 2.55 and 1.25 were obtained from Zeolyst International. Prior to ion exchange, the sodium faujasites were heated in air from room temperature to 353 K at 2.5 K min^−1^ and held at that temperature for 1 h, to 393 K at 2.0 K min^−1^ and held at that temperature for 2 h, to 823 K at 2.0 K min^−1^ and held at this temperature for 8 h. Ion-exchanged catalysts were prepared following published methods. To prepare potassium X and Y zeolites, 5 g of calcined sodium faujasite was added to 200 ml of a 2.0 M solution of KCl (≥99%, Sigma-Aldrich) in deionized water which was stirred at 298 K for 24 h. After filtration, the sample was ion-exchanged twice for 24 h in 200 ml of 1.0 M KCl [24]. Lithium-exchanged faujasites were prepared according to Feurstein [25]. Ammonium faujasites were prepared by ion exchange of sodium faujasites with two exchanges with 1.0 M and one with 2.0 M solutions of ammonium nitrate for at least 6 h at room temperature (Fisher Scientific, 99%). Sodium faujasite containing Brønsted acid sites was prepared by partial ion exchange of sodium faujasite followed by heating. Silver faujasites were prepared by ion exchange of ammonium faujasites with silver nitrate (J.T. Baker Chemical Company, 99.8%) [26]. Following the final filtration, the sample was washed three times with deionized water and dried overnight at 353 K. CuY (Si/Al = 2.55) was prepared by solid-state ion exchange (SSEI) of HY (Si/Al = 2.55) faujasite (Zeolyst International) with CuCl (Sigma Aldrich, ≥99%) [27]. Samples treated with bicarbonate salts were washed at 298 K with 0.05 M solutions for 30 min prior to calcination.

X-ray powder diffraction (XRD) patterns of the zeolite catalysts were measured using a Phillips X'Pert X-ray diffractometer with Cu K*α* radiation (*λ* = 1.5418 Å). The micropore volumes of the zeolites were calculated using the t-plot method from nitrogen adsorption isotherms measured using a Micromeritics 3-Flex instrument at 77 K. Prior to nitrogen adsorption isotherm measurement, the samples were degassed for 8 h under vacuum at 573 K. Scanning electron microscopy (SEM) and energy-dispersive X-ray spectroscopy (EDS) were performed using a JOEL JSM-7400 F high-resolution SEM. Elemental analysis is reported from inductively coupled plasma-atomic emissions spectroscopy (ICP-AES) analysis obtained from Galbraith Laboratories facility in Knoxville, TN and/or EDS. EDS results were obtained by averaging the result of three measurements of the sample. The elemental composition of Li-containing samples was measured by the disappearance of the exchanged cation. The Brønsted acid sites were characterized by temperature-programmed desorption thermal gravimetric analysis (TPD-TGA) using isopropylamine (IPA) as a probe molecule using a SDT Q600, TA thermal analyser.

### 2.2. Reaction experiments

Zeolite (0.10 g) or 0.02 g of HY (Si/Al = 2.55), 7.22 g of freshly distilled DMF (99%, Sigma-Aldrich) and 0.365 g of *n*-decane (internal standard, 99%, Sigma-Aldrich) were initially charged into a 45 ml closed Parr reactor (series 4703–4714, General Purpose Pressure Vessels). The catalyst loadings were selected such that selectivity can be compared at similar conversion. The container was then purged with nitrogen gas, pressurized with ethylene (99.5%, Air Liquide) to 4700 kPa at room temperature, and heated using a Chemglass oil-bath unit. At 493 K, the Parr reactor pressure was about 7600 kPa. Reactions run at greater than 10% conversions were run following the procedure described above except with half the amount of DMF. Experiments examining the hydrolysis of DMF reaction were run using 5.34 g of DMF, 0.5 g of deionized water and 0.365 g of *n*-decane pressurized to 280 kPa of nitrogen at room temperature. Mixing inside the reactor was accomplished by using a magnetic stirrer. Following reaction, the Parr reactor was cooled in an ice bath and the product solution was then filtered using a 0.2 µm Nalgene syringe filter. Product solutions analysed by gas chromatography (GC) were diluted in ethyl acetate (HPLC-grade, 99.9%, Sigma-Aldrich) and analysed using an Agilent 6850 series GC and 5973 MS detector. Products were confirmed by injection of standards. For product solutions analysed by ^1^H NMR spectroscopy: 0.005 ml of the product solution was diluted in 0.5 ml of deuterated dimethylsulfoxide (DMSO-d6) containing 0.03 vol. % tetramethylsilane (TMS) used as an internal standard (99.8%, Fisher). The *p*-xylene production rate per active site was calculated by dividing the moles of *p*-xylene formed per unit time by the moles of extraframework cations in the zeolite, which was determined by elemental analysis (ICP-AES).

### 2.3. Statistical analysis of reaction results: analysis of variance

ANOVA was used to test the significance of the ion-exchanged cation element and the Si/Al ratio of the faujasite zeolites on the rate of *p*-xylene production [28]. A two-factor factorial design was designed for a signal-to-noise ratio of 2.0, where the signal-to-noise ratio (*ρ*_SN_) is defined as

2.1 $$\rho_{\mathrm{SN}}=\frac{\delta^{*}}{\sigma }\text{, }$$

where δ* and *σ* represent the smallest magnitude of the effect desired to be detected and standard deviation, respectively. Based on previous data, an estimate of 0.10 was used for the standard deviation expected from experimental results [2]. A sample size of 12 was chosen based on equation 2.2 relating the signal-to-noise ratio to the appropriate sample size range:

2.2 $${\left(\frac{7}{\rho_{\mathrm{SN}}}\right)}^{2}<n<{\left(\frac{8}{\rho_{\mathrm{SN}}}\right)}^{2}.$$

The experiments were performed systematically and randomly, and analysed using the Minitab software through the Stat > DOE > Factorial > Analyze Factorial Design option.

## 3. Results

On the basis of the data obtained from batch reactor experiments and the available structural data on the extraframework positions of ion-exchanged X and Y zeolites, the structural basis for the activity and selectivity of faujasite catalysts for *p*-xylene synthesis from DMF and ethylene is reported. The discussion is supported by statistical analysis done using ANOVA of the faujasite catalysts linking properties and reaction results such as selectivity and activity obtained for the biomass-based route. Faujasite catalysts are relatively inexpensive and the solvent-free approach decreases *p*-xylene cost.

**Figure 2.**
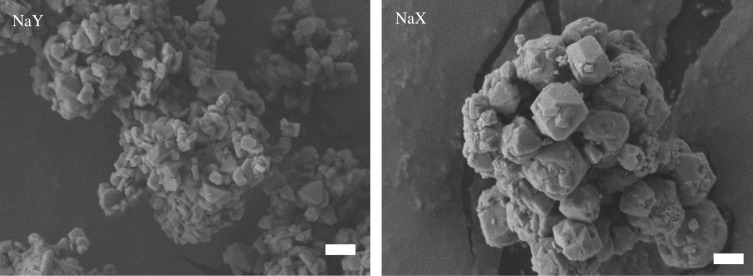
SEM images of sodium faujasite zeolites Y (Si/Al = 2.55) and X (Si/Al = 1.25). Scale bar 1 µm.

**Figure 3.**
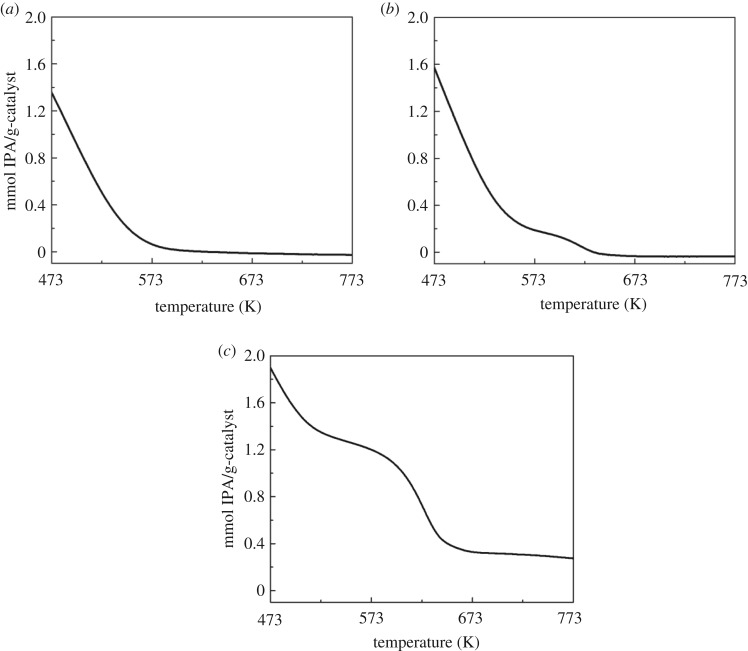
Typical TPD-TGA curve of IPA on (*a*) Na-Y, (*b*) H, Na-Y(0.1) and (*c*) H, Na-Y(0.4).

Faujasite, whose structure consists of six-membered double rings (d6R) and sodalite cages, was characterized by XRD, SEM, nitrogen adsorption, ICP-AES or EDS, and TPD-TGA to assess the structure, morphology, pore volume and chemical composition of the samples. XRD patterns show that the faujasite samples retained their crystallinity and were free of impurities and amorphous material following aqueous ion exchange (electronic supplementary material, figure S1). SEM images show that the zeolite catalyst particles have estimated individual crystallite sizes ranging approximately 0.2–2 µm in diameter and octahedral crystal habits (figure 2). The zeolite catalysts have the expected micropore volumes (approx. 0.2–0.3 cm^3^ g^−1^) of these molecular sieves following ion exchange as determined by nitrogen adsorption experiments (table 1). The micropore volumes of these samples generally decrease with increasing molecular weight of the exchanged cation with AgX at the lower end. Elemental analysis by ICP-AES or EDS shows that samples were nearly completely ion-exchanged (table 1). TPD-TGA using isopropylamine as the probe molecule indicated a two-step weight change for the ion-exchanged samples with strong Brønsted acidity (figure 3). Weight loss at low temperature (less than 573 K) is attributed to the IPA adsorbed on the weak acid sites, whereas the one between 573 K and 673 K is from the decomposition of IPA on strong Brønsted acid sites [29].

**Table 1.** Chemical compositions (Si/Al ratio) and micropore volumes of faujasite catalysts.^a^

catalyst Si/Al ratio	M/Al ratio^b^	micropore volume (cm^3^ g^−1^)
HY (44.9)	—	0.30
LiY (2.55)	0.99	0.24
LiX (1.25)	0.99	0.28
NaY (40)	0.99	0.24
NaY (2.88)	0.98	0.32
NaX (1.25)	0.99	0.27
KY (2.61)	0.92	0.28
KX (1.52)	0.99	0.25
AgY (3.17)	0.94	0.20
AgX (1.38)	1.01	0.19
CuY (3.07)	0.76	0.17

^a^The Si/Al and Si/M ratios were calculated based on EDS or the catalyst manufacturer.

^b^M/Al is defined as the atomic ratio of the ion-exchanged metal (M = Li, Na, K, Ag and Cu) versus aluminium (Al) in the sample.

The introduction of Al^3+^ into the aluminosilicate structure introduces a charge imbalance compensated by exchangeable cations which can be Brønsted or Lewis acidic. Control experiments were run to probe for residual Brønsted acidity following ion exchange. It was found that washing of the metal-exchanged faujasites (Si/Al = 2.55 and 1.25) with dilute solutions of bicarbonate salts containing the ion-exchanged metal at 298 K did not significantly affect DMF conversion or *p*-xylene yield for sodium and lithium X (electronic supplementary material, figure S2). Because basic bicarbonate solutions react with acid sites, it is concluded that the observed reactivity of the alkali metal-exchanged zeolites is owing to Lewis acid sites and not to any residual Brønsted acids sites that remain after ion exchange. For this conversion, water is formed during reaction and may affect the active site. Therefore, experiments were run monitoring the activity of the catalyst with time. The yield profile for *p*-xylene synthesis is linear with time, indicating the water formed during reaction does not affect the reaction rate or the nature of the active site (electronic supplementary material, figure S3).

The reaction of DMF and ethylene to *p*-xylene catalysed by Y and X zeolites was run in a batch reactor to establish a relationship between zeolite active site type and extraframework position on *p*-xylene production rate and selectivity. The results of these experiments such as DMF conversion, *p*-xylene selectivity and *p*-xylene production rate per active site can be found in table 2. Lewis acidic faujasite catalysts with Si/Al ratios of 40 are nearly twice as selective as Brønsted acidic HY zeolite (approx. 50 versus 30%) at similar conversion. The comparison has been made at similar conversion by using different catalyst loadings of Lewis acidic faujasite to HY to better compare selectivity. It has been observed that selectivity first decreases owing to DMF hydrolysis and then increases owing to the reversibility of this reaction [18]. As the extraframework positions shift to include sites II and III in the sodalite cage with decreasing silicon to aluminium ratio, *p*-xylene selectivity increases and activity goes through an optimum at a Si/Al ratio of 2.55 where sites I′ and II are mainly occupied. One can see how critical the effect of active site and extraframework position on selectivity can be by comparing the best Lewis acidic faujasite tested to the Brønsted HY. The optimal Lewis acid catalyst tested at 493 K, NaX zeolite, catalysed the conversion of DMF and ethylene to *p*-xylene with 75% selectivity. At the same reaction conditions, HY zeolite catalyses the reaction with 33% selectivity to *p*-xylene at similar conversions. To further increase *p*-xylene selectivity, the best catalyst was tested at a higher temperature. At 523 K, NaX catalyses the reaction with 96% selectivity to *p*-xylene at 10% DMF conversion.

**Table 2.** DMF conversion and *p*-xylene selectivity of metal-exchanged faujasites and HY zeolite for the solvent-free reaction of DMF with ethylene run at 493 K for 6 h pressurized to 4700 kPa of ethylene at 298 K.

catalyst (Si/Al ratio)	DMF conversion (%)	*p*-xylene selectivity (%)	2,5-hexanedione selectivity (%)	1-methyl-4-propylbenzene selectivity (%)	*p*-xylene production rate per active site (site^−1^ h^−1^)
HY (40)	7.5	33	—	—	4.1
LiY (40)	2.2	9.1	0.0	14	0.48
LiY (2.55)	9.5	32	5.3	31	0.84
LiX (1.25)	6.8	51	3.0	19	0.60
NaY (40)	2.3	8.7	0.0	14	0.49
NaY (2.55)	8.4	50	6.6	30	1.23
NaX (1.25)	5.2	75	5.3	3.0	0.99
KY (2.55)	10	56	22	20	1.8
KX (1.25)	4.0	48	0	0	0.40
AgY (2.55)	9.6	43	27	26	1.6
AgX (1.25)	9.5	48	13	28	1.8
CuY (2.55)	4.5	48	6.7	27	0.57

ANOVA was used to test for the statistical significance of cation type, framework composition, and the interaction between cation and framework composition on *p*-xylene production rate per active site in search of synergistic effects like those found by DFT [22,23]. *p*-values of less than 0.05 were found for cation type, framework composition and the interaction of these factors, indicating that these two factors and their interaction are statistically significant factors of the *p*-xylene production rate per active site (see the electronic supplementary material, table S1 for experimental values and run orders). Based on the statistical significance of these factors, active site type and extraframework distribution affect the rate of *p*-xylene production, which does not rule out multisite cooperativity. A main effects plot was made to determine the most important structural parameters to maximize *p*-xylene production per active site (electronic supplementary material, figure S4). To maximize *p*-xylene production per active site in the dehydration-limiting regime for catalysts tested, zeolite Y should be used with potassium cations. It is hypothesized that the reaction is run in the dehydration-limiting regime under these conditions [13]. In the Diels–Alder limiting regime, the rate of *p*-xylene synthesis is limited by the uncatalysed Diels–Alder reaction. Because the rate of *p*-xylene synthesis increases when going from Brønsted to Lewis acid-containing faujasite catalysts, it may be concluded that the conversion was run in the dehydration-limiting regime for these catalysts. The selection of the most active catalyst (KY) does not result in the best selectivity.

**Figure 4.**
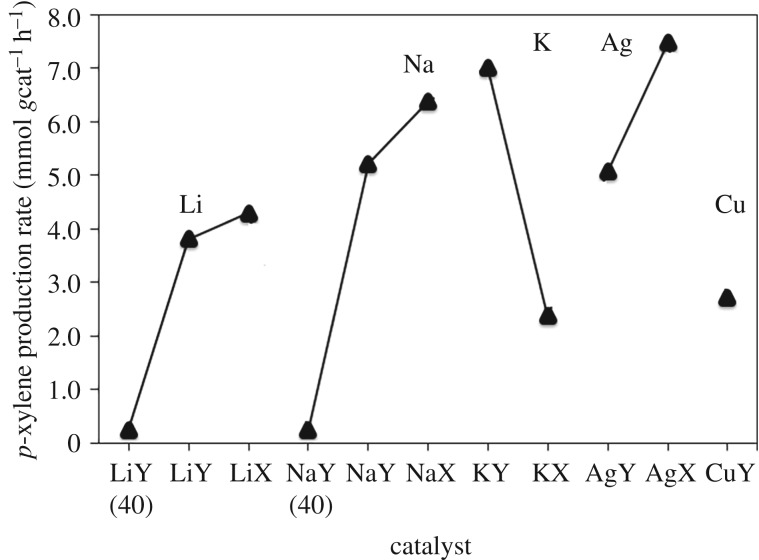
Rate of *p*-xylene synthesis per gram for Na+, Li+, K+, Ag+ and Cu+ ion-exchanged faujasites for the reaction of DMF with ethylene run at 493 K for 6 h. The overall rate of *p*-xylene production was limited by the rate of the dehydration of the Diels-Alder adduct.

**Figure 5.**
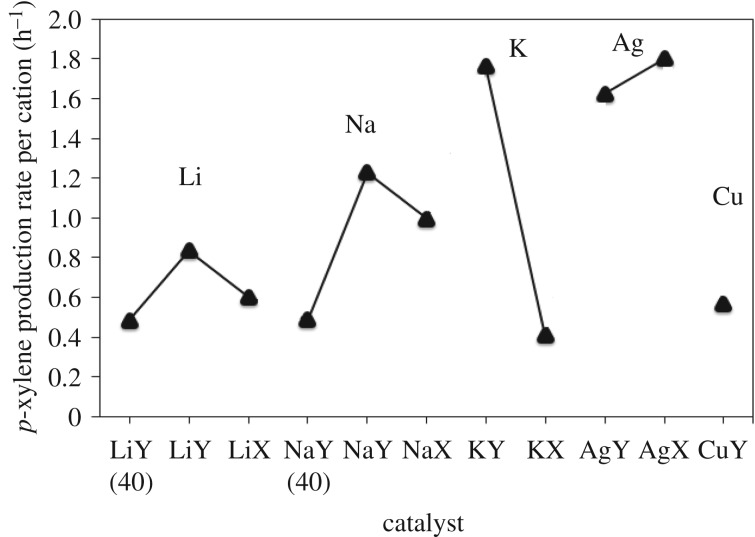
*p*-Xylene production rate per active site versus Na+, Li+, K+, Ag+ and Cu+ ion-exchanged faujasites for the reaction of DMF with ethylene run at 493 K for 6 h. The overall rate of *p*-xylene production was limited by the rate of the dehydration of the Diels-Alder adduct.

With an understanding of the statistically significant factors of the faujasite catalyst on *p*-xylene production, the rate of *p*-xylene production per gram of catalyst was determined. It was observed that decreasing the Si/Al ratio increased the rate of *p*-xylene production per gram of catalyst (figure 4). This is consistent with the hypothesis that with more active sites per gram of catalyst, the rate of *p*-xylene formation increases. This also suggests that cations in sites II and III located in the supercage also participate in the reaction. To assess the activity of each active site for *p*-xylene production, the average *p*-xylene production rate per active site was determined (figure 5). In the sodium and lithium series of alkali metal faujasites, the *p*-xylene production rate per active site increases from samples with silicon to aluminium ratios of 40 to samples where the ratio is 2.55, and then decreases again for samples with Si/Al ratios of 1.25. The observed trend is postulated to be because of the combination of effects of extraframework cation type and position in the faujasite catalyst. Potassium cations are more active than sodium cations, which are more active than lithium cations for *p*-xylene production. In comparison, the average *p*-xylene production rate per active site of HY is 4.1 cation**^−^**^1^ h**^−^**^1^, a *p*-xylene production rate per active site value more than twice that of metal-exchanged faujasites.

With an understanding of the significant factors for *p*-xylene production, attention was turned to the major by-products formed, 1-methyl-4-propylbenzene and 2,5-hexanedione, to rationalize the effect of catalyst active site and extraframework position in faujasite on by-product formation (table 2). Interestingly, selectivity to the alkylated product increases from ion-exchanged faujasites with silicon to aluminium ratios of 40 to 2.55 and decreases from metal-exchanged faujasites with silicon to aluminium ratios of 2.55 to 1.25. This may be owing to a combination of effects. Cations only occupy the less accessible site I′, located in a double six-membered ring for materials with silicon to aluminium ratios of 40. With decreasing silicon to aluminium ratios (2.55 and 1.25), extraframework cations begin to occupy the more exposed sites II and III, which become less acidic with decreasing silicon to aluminium ratio of the framework [30–32]. Therefore, it is postulated that the combination of the effects of Lewis acid strength, accessibility and extraframework position contributes to the observed selectivity trends to the by-products. Dimers and trimers were produced in less than 10% selectivity when the reaction was catalysed by NaX. On the other hand, HY catalysed the solvent-free conversion of DMF and ethylene with greater than 30% selectivity to the alkylated product, 1-methyl-4-propylbenzene and oligomers at 493 K, demonstrating how active site type (Brønsted versus Lewis) influences the rate of by-product formation. The rate of DMF hydrolysis to 2,5-hexanedione decreases when using the Lewis acidic NaY (Si/Al = 40) catalyst when compared with Brønsted acidic HY (Si/Al = 40) zeolite (DMF conversion: 1.6% versus 9.8%, 2,5-hexanedione selectivity: 75% versus 94% after 6 h of reaction at 523 K).

Additional experiments were run at 523 K to determine if the high selectivity would be maintained at greater conversions for the most promising catalyst, NaX. At greater conversions (greater than 30%), *p*-xylene selectivity of 91% was obtained under solvent-free conditions (table 3). This is better than the approximately 25% selectivity to *p*-xylene obtained by HY zeolite, 65% selectivity to *p*-xylene of H-beta in *n*-heptane, and the 60 and 80% selectivities of *p*-xylene found for Sn- and Zr-beta catalysts, respectively, in *n*-heptane solvent at the same conversion (table 3) [14,16]. Increasing the catalyst amount of NaX resulted in an increase in the rate of *p*-xylene production which is characteristic of reactions run in the dehydration-limiting regime [13,19]. Rates of irreversible Friedel–Crafts side reactions, such as alkylation and oligomer formation, are very small at higher conversions with NaX. NaX is reusable with DMF conversion and *p*-xylene selectivity maintained for two reaction cycles at 523 K, with less than a 5% drop in either conversion or selectivity. The results of this study illustrate how tuning the acidity of the active site in molecular sieves affects the experimentally observed reactivity and selectivity trends. These observations are supported by DFT and ONIOM calculations [22,23]. The greater selectivity of reaction observed when using NaX for the solvent-free conversion decreases project process cost and environmental impact.

**Table 3.** Conversion and *p*-xylene selectivity for the solvent-free Diels–Alder cycloaddition and dehydration reactions of DMF and ethylene run at 523 K for 24 h.

catalyst	mass of catalyst (g)	DMF conversion (%)	*p*-xylene selectivity (%)
NaX (Si/Al = 1.25)	2.0	30	91
HY (Si/Al = 2.25)	0.2	31	25

## 4. Conclusion

The effect of active site and extraframework position in faujasite on *p*-xylene selectivity was examined for type Y (Si/Al = 40 and 2.55) and X (Si/Al = 1.25) zeolites containing the Lewis acids Na+, K+, Li+, Ag+ and Cu+, and a Brønsted acid-containing zeolite, HY at low conversion (less than 35%). Lewis acidic faujasites were found to be more selective than Brønsted acidic faujasite. NaX, which has ions in positions II and III in the faujasite supercage, catalysed the reaction with a greater than 90% *p*-xylene selectivity at 10% conversion. Statistical analysis of the experimental results by ANOVA indicate that the optimal *p*-xylene production rate of Lewis acidic faujasite catalyst is for the KY zeolite which has extraframework positions in the d6R and supercage. For NaX, 91% *p*-xylene selectivity was obtained at 30% DMF conversion.

## Supplementary Material

Supplementary material

## Data accessibility

The datasets supporting this article have been uploaded as part of the electronic supplementary material.
