# Correction to ‘Synergestic effect of acidity and extraframework position in faujasite on renewable *p*-xylene production'

**DOI:** 10.1098/rsos.180997

**Published:** 2018-07-18

**Authors:** Eyas Mahmoud

*R. Soc. open sci*. **5**, 172471. (Published 23 May 2018). (doi:10.1098/rsos.172471)

There were a number of errors in the abstract of the published paper, the correct text is presented below:

*p*-Xylene is a commodity chemical used for the manufacture of plastic bottles and textiles. For the biomass-based route from 2,5-dimethylfuran (DMF) and ethylene, the properties of the catalyst such as acidity affect product selectivity and catalyst activity. To determine the effect of acidity and extraframework position in faujasite zeolite on *p*-xylene selectivity, type Y (Si/Al = 40 and Si/Al = 2.55) and X (Si/Al = 1.25) zeolites containing the extraframework Lewis acids Na+, K+, Li+, Ag+ and Cu+, and a Brønsted acid-containing zeolite, HY (Si/Al = 40), were prepared by ion exchange and tested for *p*-xylene production under solvent-free conditions and low conversions (less than 35%). Here, it is reported that NaX zeolite catalyses DMF and ethylene conversion to *p*-xylene with 91% selectivity at 30% conversion, which is better than the 25% *p*-xylene selectivity obtained when using HY at similar conversion. A statistical model and estimation technique, ANOVA, was used to show that there is a synergistic effect between acidity and extraframework position on the rate of *p*-xylene production. At 7% DMF conversion, Lewis acids were more selective than the Brønsted acid tested (50 versus 30% *p*-xylene selectivity). *p*-Xylene selectivity is optimal when using Lewis acids with moderate acidity and extraframework positions located in the faujasite supercage (sites II and III).

