# Supplementary material for: Synergistic effect of acidity and extraframework position in faujasite on renewable p-xylene production
Source: R Soc Open Sci. 2018 May 23;5(5):172471. doi: 10.1098/rsos.172471 (PMC5990767; doi:10.1098/rsos.172471)
Supplement: Supplementary material [file rsos172471supp1.docx]

Supplementary Material

Synergistic effect of acidity and extraframework position in faujasite on renewable *p*-xylene production

Eyas Mahmoud^†*^

^†^Department of Chemical and Petroleum Engineering, United Arab Emirates University, Al-Ain, UAE

*Author for correspondence (eyas6778@gmail.com).


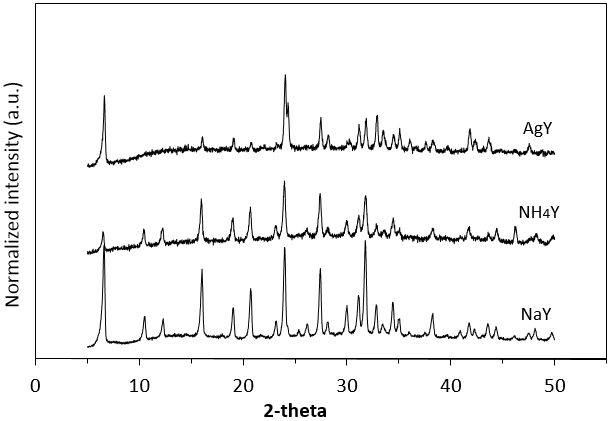


**Figure S1.** Powder XRD patterns of hydrated commercial or ion exchanged faujasite catalysts with silicon to aluminum ratios of 2.55.


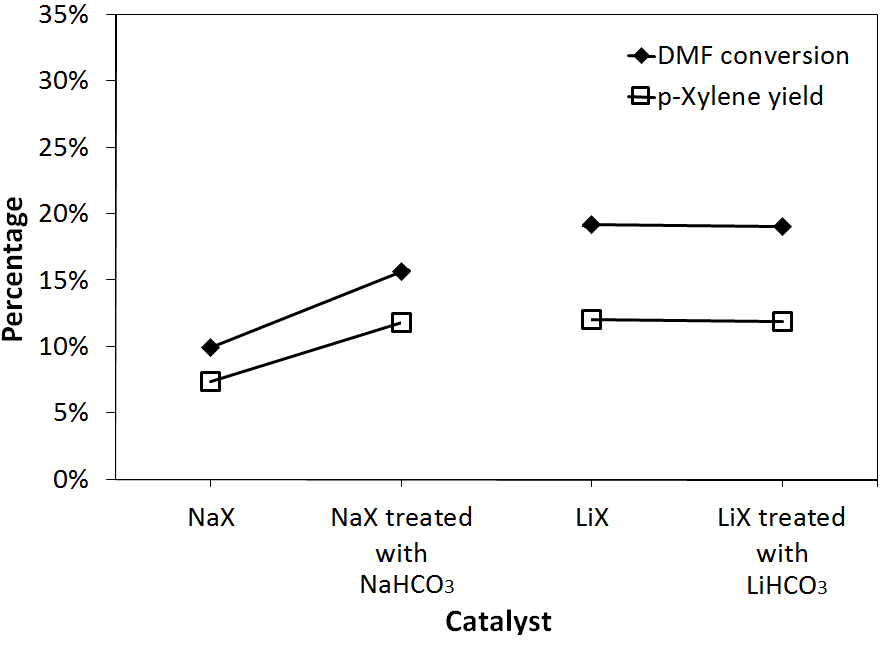


**Figure S2.** Effect of carbonate treatment on DMF conversion and *p*-xylene yield for the solvent-free reaction of DMF and ethylene at 250 °C catalyzed by NaX and LiX zeolites (Si/Al=1.25) at 4700 kPa initial pressure of ethylene at 25 °C and in the dehydration limited regime.


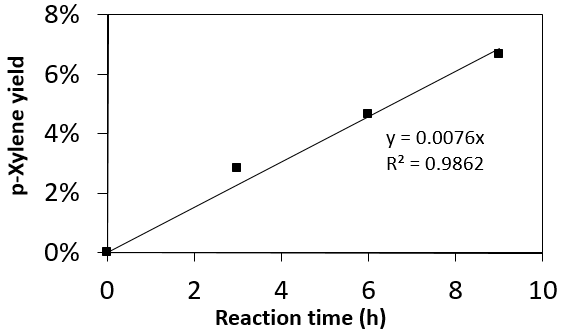


**Figure S3**. Yield profile for *p*-xylene production for the solvent-free reaction of DMF and ethylene at 250 °C catalyzed by NaX (Si/Al=1.25) zeolite at 4700 kPa initial pressure of ethylene at 25 °C and in the dehydration limited regime.

**Table S1.** *p*-Xylene production rate for a two factors experiment (cation type levels 1: Na^+^, 2: Ag^+^, 3: Li^+^ and framework type levels; 1: X and 2: Y) run with two replicates. Y zeolites have a silicon to aluminum ratio of 2.55 and X zeolites have a silicon to aluminum ratio of 1.25.

| Run order | Cation type | Framework type | Rate of *p*-xylene production (site^-1^ h^-1^) per active site |
| --- | --- | --- | --- |
| 1 | Ag | Y | 1.63 |
| 2 | Na | X | 0.904 |
| 3 | Li | X | 0.573 |
| 4 | Li | Y | 0.835 |
| 5 | Ag | X | 1.84 |
| 6 | Na | Y | 1.35 |
| 7 | Li | X | 0.629 |
| 8 | Na | Y | 1.35 |
| 9 | Na | X | 1.09 |
| 10 | Li | Y | 0.830 |
| 11 | Ag | X | 1.79 |
| 12 | Ag | Y | 1.61 |

**
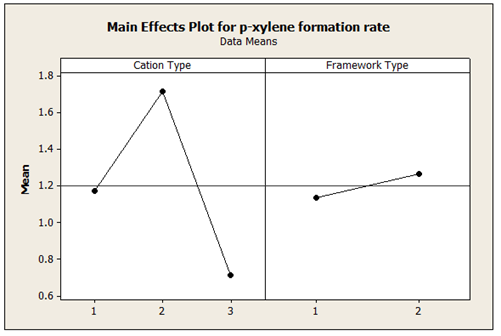
**

**Figure S4.** Main effects plot for the *p*-xylene production rate per cation. Cation type level 1: Na, 2: Ag, 3: Li; Framework type level 1: X, 2:Y.
